# No Association Between Diet Quality, Nutritional Status, and Quality of Life in Women with Hashimoto’s Thyroiditis—A Cross-Sectional Study

**DOI:** 10.3390/nu17061015

**Published:** 2025-03-14

**Authors:** Karolina Osowiecka, Damian Skrypnik, Joanna Myszkowska-Ryciak

**Affiliations:** 1 Department of Dietetics, Institute of Human Nutrition Sciences, Warsaw University of Life Sciences (WULS), 02-776 Warsaw, Poland; 2 Department of Treatment of Obesity, Metabolic Disorders and Clinical Dietetics, Poznan University of Medical Sciences, 60-355 Poznan, Poland

**Keywords:** Hashimoto’s thyroiditis, diet quality, nutrition, nutritional status, quality of life, female

## Abstract

Objectives: Dietary habits are identified as a potential factor influencing the quality of life of individuals diagnosed with Hashimoto’s disease (HAT). The objective of this study was to analyze the relationship between quality of diet and selected parameters of nutritional status, and quality of life in female patients with HAT. Methods: A descriptive cross-sectional study was conducted among 147 women aged 39.9 ± 10.39 years. Diet quality was determined with the Pro-Healthy Diet Index (pHDI-10), quality of life with a thyroid-specific questionnaire (ThyPROpl), and gastrointestinal symptoms with the Gastrointestinal Symptom Rating Scale (GSRS). Results: The mean pHDI-10 score was 25.5 ± 9.59; 80% of women had a low pro-healthy diet quality (LQD group, 21.9 ± 6,89 pts.) and 20% had a medium pro-healthy diet quality (MQD group, 39.7 ± 4.69 pts.). Nutritional status and anthropometric and health risk parameters did not differ between the groups. The ThyPROpl score was 49.15 ± 31.16 (LQD: 49.58 ± 31.01, MQD: 47.41 ± 32.28, *p* = 0.73). Conclusions: Quality of diet was not associated with the quality of life or nutritional status of HAT patients. However, the majority of participants showed poor healthy eating habits, elevated body mass index and unsatisfactory quality of life in areas, such as tiredness, hypothyroid symptoms, depression, emotional vulnerability, and anxiety.

## 1. Introduction

It is estimated that thyroid disease affects 9.5% of the Polish population. Thyroid disease exhibits a female-to-male ratio of approximately 6.3:1 [1]. The majority of individuals with thyroid disease are affected by Hashimoto’s autoimmune thyroiditis (HAT), which also exhibits a pronounced female-to-male predominance, with a ratio of approximately 4:1 [2]. The etiology of Hashimoto’s disease is complex and not yet fully understood. The development of HAT may be triggered and/or increased by a number of factors, including genetic and environmental factors (e.g., dietary factors, smoking, bacterial or viral infections, exposure to certain chemical components) and may coexist with other autoimmune diseases, including type 1 diabetes, coeliac disease, psoriasis, rheumatoid arthritis, and multiple sclerosis [3].

The consequence of an abnormal immune response and morphological alterations in the thyroid gland is a gradual deterioration in its functionality. In conditions of thyroid hormone deficiency and the subsequent weakening of their action at the cellular level, the metabolic rate is observed to decrease. Additionally, insulin resistance is developed in the liver and peripheral tissues, and changes are noted in the course of carbohydrate and lipid metabolism pathways [3]. The presence of symptoms in individuals with HAT is related to the progression to hypothyroidism. The most commonly reported symptoms include weakness; chronic fatigue; anemia; sleep problems; emotional changes; excessive sweating; swelling of the face, hands and feet; feeling cold; weight gain; constipation; dry skin; and hair loss [4]. All of these symptoms of thyroid dysfunction can have a negative impact on an individual’s quality of life.

Nevertheless, even in the absence of overt thyroid dysfunction, individuals with Hashimoto’s disease have been observed to experience a reduction in quality of life [5]. This may be attributed to the presence of antibodies against thyroid peroxidase (anti-TPO) and thyroglobulin (anti-TG), which negatively affect the thyroid gland [6].

Some studies indicate that antibodies can have a detrimental impact on psychological well-being and cognitive function [6,7,8]. Bektas Uysal and Ayhan [5] also observed increased irritability and impaired physical functioning. Furthermore, there is evidence that long-term treatment with levothyroxine (LT4), the typical therapy used in HAT, may also have a negative impact on cognitive function [7].

In the existing literature, dietary habits are identified as a potential factor influencing the quality of life of individuals diagnosed with Hashimoto’s disease [5]. The scientific literature attests to the beneficial effects of healthy dietary patterns, such as the anti-inflammatory Mediterranean diet, on overall mental and physical health [9]. In contrast, a dietary pattern characterized by high consumption of processed foods may promote pro-inflammatory effects and contribute to the development of health outcomes such as obesity, metabolic disease, constipation, and cognitive and mental disorders [10]. Furthermore, the Western dietary model may also be associated with elevated levels of anti-TPO or anti-TG antibodies [11]. Additionally, obesity, a prevalent consequence of the Western diet, has been linked to elevated anti-TPO antibody levels. This may be attributable to an excess of body fat, which has been demonstrated to have pro-inflammatory effects [12]. Currently, there is no uniform position or standardized approach to dietary management in Hashimoto’s disease [13]. Furthermore, there is a paucity of high-quality research examining the efficacy of specific dietary interventions [14]. Consequently, patients may elect to adhere to dietary treatments that have not been empirically validated [15] or have only been demonstrated to be efficacious in a limited number of cases [16]. Elimination diets, in particular, have a detrimental impact on nutritional status and may increase the level of stress associated with the necessity to adhere to the dietary regimen [17].

The objective of this study was to assess the relationship between quality of diet and selected parameters of nutritional status, and quality of life in female patients with HAT. The following hypotheses have been formulated: 1. Adherence to a healthy diet is associated with a healthier body mass index in a group of women with HAT. 2. Adherence to a healthy diet is associated with a better quality of life in a group of women with Hashimoto’s disease. To the best of our knowledge, no studies have been conducted in this area in Poland. Thus, our findings may contribute to the understanding of whether diet (and/or specific dietary elements) are associated with selected aspects of nutritional status and quality of life in patients with HAT which may translate into more effective dietary recommendations for this group of patients.

## 2. Materials and Methods

### 2.1. Study Design and Selection of Participants

A descriptive cross-sectional study was conducted among adult Caucasian females who had been diagnosed with Hashimoto’s thyroiditis. The study population was recruited using the snowball sampling method between 2022 and 2023. The recruitment process was primarily conducted via social networking sites, and invitations were also sent to available medical facilities and pharmacies. The sample was selected purposively and according to the following inclusive criteria: female, medical diagnosis of Hashimoto’s disease based on anti-TPO, anti-TG, or a characteristic ultrasound image for HAT, thyroid function status (euthyroid), and age in the range of 18–64 years. Individuals with thyroid diseases other than HAT, those pregnant or breastfeeding, or those diagnosed with cancer were excluded from the study, as were any other individuals for whom participation in the study would not be in their best medical interest. In case patients exhibited contraindications to body composition analysis (e.g., the presence of metal elements within the body), this stage of the study was excluded. Data were collected using two methods: CAWI (computer-assisted web interview) and PAPI (paper-and-pencil personal interview). Participation in the study was voluntary, and written informed consent was obtained from all participants after they were informed of the purpose and procedure of the study. The study was conducted in accordance with the Declaration of Helsinki and the protocol was approved by the Ethics Committee of the Institute of Human Nutrition, Warsaw University of Life Sciences WULS, Poland (No. 22/2021, No. 21/2022).

### 2.2. Diet Quality

The Polish-validated questionnaire Pro-Healthy Diet Index (pHDI–10) [18] was used to assess the overall pro-healthy diet quality of the participants. The pHDI–10 includes ten food items: wholemeal bread/rolls, coarse-grained cereals, milk, fermented milk beverages, fresh cheese curd products, white meat, fish, pulse-based foods, fruit, and vegetables. Based on each individual’s answers to the questions, the total score is recalculated into a range of 0–100 pts. according to the questionnaire instructions. The higher the value of the index, the higher the intensity of the beneficial characteristics for health (low: 0–33 pts.; medium: 34–66 pts.; high: 67–100 pts.) [18]. The pHDI-10 internal reliability is 0.66–0.71, with a good reproducibility (kappa values 0.43–0.80) [19].

### 2.3. Nutritional Status

The anthropometric measurements were taken in accordance with the established standard procedure [20]. Body weight was determined by means of an electronic digital scale, with the subject standing. The individual undergoing examination was weighed in undergarments without footwear, with the recorded values having an accuracy of 100 g. Body height was determined with the use of a medical scale equipped with a built-in height gauge, in accordance with the standardized standing anthropometric position (lower limbs straightened, feet set parallel to each other, upper limbs straightened, hanging loosely along the trunk, headset in the eye–ear plane), with the subject barefoot. The measurements were recorded with an accuracy of 1 mm. The waist circumference was measured at the midpoint between the inferior margin of the least palpable rib and the superior aspect of the iliac crest. This was performed in a standing position with the body weight distributed evenly on both feet, after emptying the bladder and with relaxed muscles, with an accuracy of 1 mm. The circumference of the hips was measured at the widest point of the buttocks, in a standing position with the body weight distributed evenly on both feet, after emptying of the bladder and with muscles relaxed. The measurement was taken to the nearest 1 mm.

Based on anthropometric data, body mass index (BMI = weight [kg]/(height [m])^2^) [21], waist-to-hip ratio (WHR = waist circumference [cm]/hip circumference [cm]) [22], and waist-to-height ratio (WHtR = waist circumference [cm]/height [cm]) were calculated [23]. Overweight and obesity were identified as a BMI between 25.0 and 29.9 kg/m^2^ and BMI ≥ 30.0 kg/m^2^, respectively. Abdominal obesity was identified as WHR ≥ 0.80 [22]. The WHO-recommended women-specific waist circumference cut-off points for increased and substantially increased risk of metabolic complications were adopted (>80 cm and >88 cm, respectively) [22]. Based on WHtR, no increased health risk (<0.5), increased risk (0.5 ≥ WHtR < 0.6), or very high risk (≥0.6) [23] was identified.

The body composition of the subjects, comprising muscle mass, total fat, and water content, was analyzed using the bioelectrical impedance (BIA) method with a body composition analyzer (ACCUNIQ BC-720) (Selvas Healthcare, Daejeon, Republic of Korea). The measurement was conducted following the procedure specified by the manufacturer of the measuring apparatus.

### 2.4. Blood Pressure

Peripheral blood pressure was measured using the ACCUNIQ BC-250 device (Selvas Healthcare, Daejeon, Republic of Korea). Prior to the measurement, the patient was seated in a quiet room at an optimal temperature. The patient was required to sit in a seated position for a minimum of five minutes and to abstain from consuming coffee, smoking cigarettes or taking other stimulants for a minimum of 30 min prior to measurement [24].

### 2.5. Quality of Life

The participants’ quality of life was evaluated using a thyroid-specific questionnaire linguistically validated for use with the Polish population (ThyPROpl) [25]. The questionnaire comprises 85 questions, which are grouped into 13 scales that assess various aspects of quality of life that are pertinent to individuals with thyroid disorders. The 13-point scale encompasses a series of questions pertaining to a range of domains, including the following: (1) symptoms; (2) feeling tired; (3) energy; (4) memory and concentration; (5) nervousness and tension; (6) mental well-being; (7) problems with coping or mood swings; (8) relationships with other people; (9) daily activities; (10) sex life; (11) the impact of the disease or its treatment on appearance; (12) the intensity of the impact of the disease on the subject in general. The respondents were required to select one of the five available answer categories ranging from “not at all” to “very much”. The score was calculated on a scale of 0 to 100, with higher scores indicating a poorer quality of life. Reported test–retest reliability for the ThyPRO was 0.77–0.89 [26].

As the second tool to assess participants’ health-related quality of life, the Gastrointestinal Symptom Rating Scale (GSRS) was used [27]. This disease-specific questionnaire comprises 15 items combined into five symptom clusters depicting Reflux, Abdominal pain, Indigestion, Diarrhea, and Constipation. A seven-point graded Likert-type scale is used, where 1 represents the absence of troublesome symptoms and 7 represents very troublesome symptoms. The GSRS has been validated for the Polish population [28]. Cronbach’s alpha coefficient value at the first completion of the questionnaire was 0.58–0.88, and in the test–retest reliability study, it was 0.34–0.63.

### 2.6. Statistical Analyses

All statistical analyses were conducted using the software package Statistica 13.1 PL (StatSoft Inc., Tulsa, OK, USA; StatSoft, Krakow, Poland). Prior to performing statistical analysis, the normality of the distribution of variables was evaluated using the Shapiro–Wilk test. For variables with a normal distribution, a *t*-test was employed; for variables without a normal distribution, the Mann–Whitney test was applied. The effect sizes of Mann–Whitney U tests were determined with the Glass rank biserial coefficient (rg) and the Hedges g was used for *t*-tests. The Pearson chi-squared test was used to ascertain significant differences for categorical variables. Cramer’s V was used to assess the strength of association between two categorical variables. *p*-values less than 0.05 were considered statistically significant.

## 3. Results

A total of 147 women, with an average age of 39.9 ± 10.39 years and an average BMI of 26.2 ± 5.18 kg/m^2^, were enrolled in this study. The mean pHDI-10 score was 25.5 ± 9.59 pts. (Supplementary Materials, Table S1). Based on this score, 118 (80%) of the participants were classified as having a low pro-healthy diet quality (LQD), while 29 (20%) were placed in a group for medium pro-healthy diet quality (MQD). None of the patients belonged to the group with a high pro-healthy diet quality.

Table 1 presents the sociodemographic, anthropometric, and health characteristics of all the study participants, as well as those belonging to the groups of low and medium pro-healthy diet quality. No statistically significant differences were identified in the examined parameters between the groups.

Table 2 presents the body weight status and the associated health risks of all examined women, classified according to the quality of their diet. More than half of the group was characterized by excess body weight, central obesity and an increased risk of diseases based on WHtR and waist circumference. No significant differences were observed between women with low and medium pro-heathy diet quality in the assessed parameters.

Table 3 presents data on quality of life and the severity of gastrointestinal symptoms. Quality of life based on ThyPROpl indicates that the symptoms are experienced by the patients moderately. No significant difference was observed between overall quality of life and pro-healthy diet quality. The participants experienced tiredness to the greatest extent, followed by hypothyroid symptoms, depression, emotional susceptibility and anxiety. On the other hand, they assessed goiter symptoms and impaired social life to the least negative extent. The results of the analyzed quality-of-life domains did not differ between patients with LQD and MQD. Among the gastrointestinal symptoms based on the GSRS score, the most bothersome was indigestion followed by constipation and abdominal pain. The severity of any of the assessed gastrointestinal symptoms did not differ between women with LQD and MQD.

## 4. Discussion

This study does not demonstrate a significant relationship between the extent of adherence to a healthy diet and the nutritional status and quality of life of patients with HAT. However, the vast majority of participants exhibited low health-promoting eating habits, with not a single respondent demonstrating a high-quality diet. According to extensive literature data, such dietary habits may result in a significant risk of obesity [29,30], particularly central obesity [31].

According to the National Health Fund, in 2019, 29% of Polish women were found to be overweight, while 21% were obese. In the year 2023, the Supreme Audit Office reported that between 59% and 69% of the Polish population was affected by overweight and obesity, depending on the province in Poland [32]. Obesity leads to the development of a multitude of diseases, e.g., cardiovascular, metabolic, and psychological disorders such as depression or anxiety, and above all, it shortens life expectancy. At the present time, there is sufficient knowledge to determine whether obesity has an impact on HAT or whether HAT has an effect on obesity, and the relationship seems to be mutual. Excessive TSH production has been demonstrated to promote weight gain. Conversely, obesity has been shown to affect the development of HAT by producing pro-inflammatory adipokines and mediators by adipocytes, contributing to the development of inflammation. Obesity can also lead to gut dysbiosis, which can promote the production of pro-inflammatory cytokines and Th1 and Th17 lymphocytes. This effect promotes autoimmune diseases, including HAT [33,34].

In this study, the prevalence of excess body weight among women exceeded 50%. This finding is consistent with other studies that have also reported a high proportion of overweight individuals among Hashimoto thyroiditis patients [34,35,36]. In our study, both the mean BMI and the median BMI of the patients indicated overweight, and this finding is confirmed by other authors [11,35,36,37,38,39,40]. However, in some studies, the mean and/or median BMI of individuals with HAT was found to be close to normal [41,42,43,44]. The percentage of body fat measured in this study corresponded to the results obtained in other studies [35,36,45], in which the body fat percentage was higher in the HAT group than in the control [35,45]. As for the total body water content, in the study by Malczyk et al., it was lower in individuals with HAT than in healthy people [35]. In some studies, it was shown that the mean or median WHR in patients with HAT indicated abdominal obesity [36,42], but not in all studies [38,39]. WHR in patients with HAT did not differ from the control group [38,42] or was significantly lower [36]. A waist circumference above 80 cm increases the risk of metabolic diseases [22]. In our study, over 60% of examined women were at risk of metabolic diseases. Similarly, an average waist circumference above 80 cm was observed in the study by Mikulska-Sauermann et al. [36]. The mean and median systolic and diastolic blood pressure in our study did not indicate hypertension, which was also consistent with other studies [39,41,43,46,47,48]. Systolic and diastolic blood pressure were lower in HAT than in the control [43]. In another study, systolic and diastolic blood pressure did not differ [46]. Hypertension was observed in 9.6% [37] and 32.9% of patients with HAT [34].

The present study found no relationship between diet quality and quality of life. However, the extant literature indicates that poor health-promoting eating habits may result in a diminished quality of life, as evidenced in the neuropsychiatric field [49,50,51,52,53,54,55,56] and in other health consequences [30,57,58]. A significant proportion of patients diagnosed with HAT (or subclinical hypothyroidism) exhibited a lower overall quality of life in comparison to the control subjects [5,8,44,59], despite achieving normal thyroid hormone levels (euthyroid) with LT4 [59,60]. Thus, reduced quality of life may be related to thyroid autoimmunity [6,47]. This observation may imply that the disease itself exerts such a profound adverse effect on quality of life that the influence of diet alone may be negligible.

In the present study, patients reported fatigue (tiredness), decreased overall quality of life, depression, symptoms of hypothyroidism, emotional vulnerability, and anxiety as the symptoms they found most bothersome. Conversely, goiter and impaired social life were reported as the least bothersome symptoms. Tiredness was also reported as one of the most bothersome symptoms in HAT patients in other studies [44,61], in a meta-analysis on subclinical hypothyroidism [59] and in a study among patients with thyroid disease despite treatment levothyroxine and independently of hormones (TSH, FT4) [62]. Additionally, a positive correlation was observed between fatigue and anti-TPO and anti-TG antibodies [44]. In the meta-analysis by Siegmann et al., the risk of anxiety disorders was 2 times higher, and in the case of symptoms of depression, the risk was 3.5 times higher in patients with hypothyroidism in autoimmune thyroiditis [63]. According to a meta-analysis by Patti et al., quality of life, including mental health, i.e., depression or anxiety disorders, may be associated with thyroid antibodies [8]. The severity of anxiety or depressive symptoms may also result from the influence of TSH and thyroid hormones [61,64], although not always [62,65]. LT4 treatment also did not improve these symptoms [62,65]. Emotional vulnerability was also one of the main symptoms in the study by Al Quran et al. and was not associated with TSH or FT4 [62]. According to the meta-analysis by Patti et al., there is a rather unlikely association between thyroid antibodies and cognitive functions, but this requires further research [8].

However, the smallest impact of goiter symptoms (including hoarseness, a lump in the throat, difficulty swallowing, visible swelling in front of the neck [28]) was confirmed by the study by AI Quran et al., in which an additional relationship was observed between this symptom and TSH and FT4 [62]. However, the systematic review by Yuan et al. [66] and the meta-analysis by Yue et al. [67] emphasize the significant impact of goiter and larynx symptoms on the quality of life of patients with HAT. This may manifest itself through, among others, a change in voice or throat discomfort [66,67]. In Barić’s study, higher anti-TG was associated with a rougher voice [47]. Before thyroidectomy, patients with HAT compared to patients with benign goiter had worse symptoms of hypothyroidism [61]. Higher anti-TG was associated with greater edema of the eyes [47]. Anti-TG antibodies were inversely correlated with social life [44]. Before thyroidectomy, patients with HAT compared to patients with benign goiter had worse symptoms of sexual life [61]. Patients with euthyroid HAT had more skin problems, i.e., dry skin, itching, and hair loss. The higher the anti-TPO titter, the drier the skin, and the higher the anti-TG, the more severe the mucocutaneous symptoms were [44]. Higher anti-TG was also associated with worse hair condition [47].

With regard to gastrointestinal complaints, the study revealed that indigestion was the most bothersome, and reflux symptoms the least so. In Li’s study, patients with HAT in euthyroid experienced more constipation, flatulence and diarrhea compared to the control group [44]. Additionally, they showed that the higher the titter of anti-TPO and anti-TG, the more severe the abdominal distension and diarrhea were [44]. The literature shows that the hypothalamic–pituitary–thyroid axis affects the functioning of the gastrointestinal tract by regulating motility and the rate of secretion of digestive enzymes [68]. Hypothyroidism has been demonstrated to be associated with constipation, while hyperthyroidism has been shown to be linked to diarrhea [44]. Among the common gastrointestinal disorders in hypothyroidism that hinder the effectiveness of levothyroxine treatment include lactose intolerance and Helicobacter pylori [69]. In Li’s study, despite euthyroid, fT3 levels were correlated with chronic diarrhea in the general population and fT4 levels with diarrhea in women aged 40–60 years old [44]. Another study found that free T3 was positively correlated with chronic diarrhea and the number of bowel movements, and for fT4 above 0.79 ng/dL, there was a nonlinear relationship with chronic diarrhea [70].

In our study, 80% of the women had a low health-promoting diet. Other studies have also observed that the eating habits of individuals with HAT deviate from a healthy, anti-inflammatory, Mediterranean dietary pattern [34,40,42]. It was assessed that the higher the adherence to the Mediterranean diet, the lower the anti-TPO and the greater protection against the risk of developing thyroid autoimmunity [42]. In another study, healthier dietary habits had a beneficial association with thyroid function, but the association with thyroid antibodies was not significant [71]. The dietary inflammation index was associated with the hormones TSH [34,38,40] and fT4 [40]. In the case of anti-TPO, its concentration was twice as high, but not significantly higher, with a high pro-inflammatory diet [40]. In another study, the inflammation index in the diet was positively correlated with anti-TPO and anti-TG. In turn, total antioxidant capacity correlated negatively with antibodies [38].

Low oxidative stress was associated with eating vegetables at least 7 times a week and fruits at least 14 times a week [39]. In our study, no relationship was observed between the quality-of-life domains and individual groups of food products. Kaličanin et al. only noted a correlation between fruit consumption and constipation (among 16 hypothyroidism symptoms studied) [43]. Vegetables, legumes, nuts and muesli consumption was negatively associated with anti-TPO and/or anti-TG concentration [11]. The better the quality of the diet was in terms of anti-inflammatory properties, the more nutritious it was [40], although not always linearly [38,72,73]. There are a number of publications on the influence of ingredients on HAT, especially iodine, selenium, zinc, vitamin D and iron, which affect not only the functioning of the thyroid gland but also the symptoms [13,74,75,76,77,78,79,80]. Iron in particular is associated with fatigue [72], which was reported by most people with HAT in our study. According to the study by Zhang et al., iron is the most significant variable influencing HAT [37]. Recent studies also show the effect of vitamin B_12_ and magnesium on thyroid antibodies [81,82,83,84].

This study is significant for several reasons. The study provides the first knowledge about nutrition, nutritional status, and quality of life in HAT, with previous nutritional studies in HAT lacking references to quality of life. It should be further noted that a significant number of people were in attendance.

However, the study has several limitations. Firstly, a significant disparity is evident in the number of individuals across the two groups, with a preponderance of those with a low-pro-healthy-diet-quality relative to those with a medium pro-healthy quality diet. Furthermore, the present study revealed that none of the subjects had a high-quality diet. Given the size of the group, this may be indicative of poor nutrition quality for women suffering from Hashimoto’s disease. Research conducted by other Polish authors indicates that the quality of women’s diets is low, which is even more evident in women with diagnosed diseases compared to healthy women (mean pHDI score 22.3 vs. 24.7, respectively) [19]. Conversely, the underlying cause may be attributed to the questionnaire’s design, which is intended for a general population of individuals deemed to be healthy and not in need of a therapeutic diet. As with all questionnaire-based studies, the obtained data depend on the accuracy and honesty of the patient. However, all participants were allotted sufficient time to complete the questionnaires, and in the event of any uncertainty, they were encouraged to consult the researcher. Participants were requested to provide sincere responses to the questions posed.

## 5. Conclusions

This study does not demonstrate a significant relationship between the degree of adherence to a healthy diet and the nutritional status and quality of life in patients with HAT. However, the vast majority of participants showed poor healthy eating habits, abnormal BMI, and unsatisfactory quality of life in many areas. Further studies are required, including interventional studies, to more clearly determine the impact of diet on the course of HAT and especially quality of life. Additionally, there is a need to develop a dietary quality index specific to patients with HAT, who may have different nutritional needs than healthy individuals.

## Figures and Tables

**Table 1:** The demographic, anthropometric, and health characteristics of the participants (*n* = 147).

Indicator	Total (*n* = 147) Mean ± SD (Median; Q1–Q3)	LQD (*n* = 118) Mean ± SD (Median; Q1–Q3)	MQD (*n* = 29) Mean ± SD (Median; Q1–Q3)	*p*-Value	Effect Size
Age [years]	39.9 ± 10.39 (40.0; 32.0–48.0)	40.3 ± 10.39 (40.5; 32.0–48.0)	38.0 ± 10.43 (40.0; 30.0–46.0)	0.30 ^1^	0.22 ^3^
BMI [kg/m^2^]	26.2 ± 5.18 (25.7; 22.03–29.79)	26.0 ± 5.21 (25.4; 21.94–29.79)	26.8 ± 5.08 (26.6; 23.10–29.07)	0.52 ^2^	−0.05 ^4^
Waist circumstance [cm]	87.2 ± 13.98 (87.5; 76.5–98.5)	87.0 ± 14.06 (87.7; 75.0–99.0)	87.5 ± 13.89 85.0; 77.0–95.0)	0.89 ^2^	0.01 ^4^
Hip circumstance [cm]	105.4 ± 9.90 (104.0; 99.0–112.0)	105.3 ± 10.01 (105.0; 98.0–112.0)	106.1 ± 9.48 (102.0; 100.0–114.5)	0.97 ^2^	−0.03 ^4^
WHR	0.82 ± 0.08 (0.82; 0.75–0.89)	0.83 ± 0.09 (0.82; 0.75–0.89)	0.82 ± 0.07 (0.82; 0.78–0.86)	0.96 ^2^	0.01 ^4^
WHtR	0.53 ± 0.09 (0.52; 0.45–0.59)	0.52 ± 0.09 (0.52; 0.45–0.59)	0.53 ± 0.09 (0.52; 0.45–0.56)	0.98 ^2^	−0.01 ^4^
Fat mass [%] *	33.6 ± 7.84 (34.7; 27.4–39.5)	33.2 ± 8.09 (34.4; 26.2–39.6)	35.2 ± 6.53 (35.4; 30.6–38.3)	0.34 ^2^	−0.05 ^4^
Fat-free mass [kg] *	47.2 ± 6.04 (46.8; 43.1–50.9)	47.2 ± 6.16 (46.8; 43.3–50.9)	47.0 ± 5.57 (46.9; 42.2–49.6)	0.86 ^1^	0.03 ^3^
Muscle mass [kg] *	34.6 ± 4.42 (34.2; 31.5–37.1)	34.6 ± 4.51 (34.2; 31.7–37.2)	34.4 ± 4.03 (34.4; 30.9–36.2)	0.84 ^1^	0.02 ^3^
Total body water [L] *	26.3 ± 3.38 (26.1; 24.0–28.3)	26.3 ± 3.45 (26.1; 24.1–28.3)	26.2 ± 3.09 (26.2; 23.5–27.6)	0.84 ^1^	0.02 ^3^
Systolic pressure [mmHg]	122.7 ± 15.59 (122; 113–130)	123.6 ± 16.03 (122; 113–131)	119.3 ± 13.45 (117; 110–129)	0.21 ^2^	0.10 ^4^
Diastolic pressure [mmHg]	72.5 ± 10.94 (73; 64–79)	72.9 ± 11.35 (73; 64–79)	70.5 ± 9.01 (73; 64–76)	0.27 ^2^	0.08 ^4^
Pulse [bpm]	71.5 ± 9.37 (70; 66–77)	71.9 ± 11.35 (70; 66–78)	69.6 ± 6.69 (70; 64–75)	0.36 ^2^	0.07 ^4^

^1^ Student *t*-test; ^2^ Mann–Whitney test; LQD—low-pro-healthy-diet-quality group; ^3^ Hedges g coefficient; ^4^ Glass rank biserial coefficient; MQD—medium-pro-healthy-diet-quality group; bpm—beats per minute; * *n* = 134.

**Table 2:** The participants’ body weight status and health risk (*n* = 147).

Parameter	Total *n* (%)	LQD (*n* = 118) *n* (%)	MQD (*n* = 29) *n* (%)	*p*-Value ^1^	Cramer’s V
Body weight status based on BMI					
Underweight	5 (3.4)	5 (4.2)	0 (0)	0.57	0.12
Normal	63 (42.9)	52 (44.1)	11 (37.9)		
Overweight	46 (31.3)	35 (29.7)	11 (37.9)		
Obesity	33 (22.4)	26 (22.0)	7 (24.1)		
WHR					
Abdominal obesity (≥0.80)	59 (40.1)	48 (40.7)	11 (37.9)	0.78	0.02
No abdominal obesity (<0.80)	88 (59.9)	70 (59.3)	18 (62.1)		
Risk of metabolic complications based on WC				
Small/normal (≤80 cm)	51 (34.7)	40 (33.9)	11 (37.9)	0.87	0.04
Increased (>80–88 cm)	29 (19.7)	23 (19.5)	6 (20.7)		
Substantially increased (>88 cm)	67 (45.6)	55 (46.6)	12 (41.4)		
Cardiometabolic risk based on WHtR				
No increased risk (<0.5)	63 (42.9)	50 (42.4)	13 (44.8)	0.94	0.03
Increased risk (0.5–0.59)	50 (34.0)	40 (30.9)	10 (34.5)		
Very high risk (≥0.6)	34 (23.1)	28 (23.7)	6 (20.7)		

^1^ chi-square test; LQD—low-pro-healthy-diet-quality group; MQD—medium-pro-healthy-diet-quality group; BMI—body mass index; WHR—waist-to-hip ratio; WC—waist circumference; WHtR—waist-to-height ratio.

**Table 3:** Quality of life and severity of gastrointestinal symptoms of participants (*n* = 147).

Parameters	Total (*n* = 147) Mean ± SD (Median; Q1–Q3)	LQD (*n* = 118) Mean ± SD (Median; Q1–Q3)	MQD (*n* = 29) Mean ± SD (Median; 13)	*p*-Value ^1^	Effect Size ^2^
Quality of life (ThyPROpl)					
Goiter symptoms	17.78 ± 17.23 (11.36; 4.55–27.27)	17.58 ± 16.08 (11.36; 6.18–27.27)	18.57 ± 21.60 (11.36; 4.55–22.73)	0.59	0.04
Hyperthyroid symptoms	31.56 ± 19.41 (28.12; 15.63–43.75)	31.48 ± 17.22 (29.69; 15.63–43.75)	31.89 ± 26.91 (25; 9.38–37.50)	0.36	0.05
Hypothyroid symptoms	44.89 ± 24.45 (43.75; 25.00–62.50)	44.54 ± 23.64 (43.75; 25.00–62.50)	46.34 ± 27.93 (43.75; 25.00–68.75)	0.83	−0.02
Eye symptoms	25.57 ± 21.34 (18.75; 9.38–37.50)	26.11 ± 20.46 (18.75; 9.38–37.50)	23.38 ± 24.88 (15.63; 3.13–34.38)	0.22	0.10
Tiredness	64.75 ± 19.89 (64.29; 50.00–78.57)	63.77 ± 20.19 (64.29; 46.43–78.57)	68.72 ± 18.35 (75.00; 57.14–78.57)	0.24	−0.09
Cognitive complaints	37.79 ± 22.44 (37.50; 20.83–50.00)	38.07 ± 22.96 (37.5; 20.83–50.00)	36.64 ± 20.52 (37.50; 25.00–50.00)	0.89	0.01
Anxiety	40.96 ± 22.54 (41.67; 25.00–58.33)	39.69 ± 21.95 (37.50; 25.00–58.33)	46.12 ± 24.52 (50.00; 20.83–66.67)	0.20	−0.01
Depressivity	44.51 ± 22.52 (42.86; 28.57–60.71)	43.75 ± 21.76 (42.86; 28.57–60.71)	47.66 ± 25.56 (42.86; 28.57–60.71)	0.49	−0.06
Emotional susceptibility	43.69 ± 22.13 (41.67; 27.78–61.11)	42.96 ± 21.76 (38.89; 27.78–61.11)	46.65 ± 23.74 (41.67; 25.00–66.67)	0.44	−0.06
Impaired social life	19.86 ± 12.50 (12.50; 0.00–31.25)	18.96 ± 17.73 (12.50; 0.00–31.25)	23.49 ± 24.59 (12.50; 0.00–50.00)	0.64	−0.04
Impaired daily life	23.58 ± 21.25 (20.83; 4.17–37.50)	23.45 ± 20.57 (20.83; 8.33–37.50)	24.14 ± 24.19 (16.67; 4.17–33.33)	0.79	0.02
Impaired sex life	36.22 ± 31.71 (25.00; 0.00–62.50)	36.76 ± 31.11 (20.83; 0.00–62.50)	34.05 ± 34.54 (37.5; 0.00–62.50)	0.56	0.05
Cosmetic complaints	35.03 ± 25.29 (3.33; 12.50–54.17)	35.38 ± 25.12 (33.33; 12.50–54.17)	33.62 ± 26.37 (37.50; 12.50–54.17)	0.65	0.04
Overall quality of life	49.15 ± 31.16 (50.00; 25.00–75.00)	49.58 ± 31.01 (50.00; 25.00–75.00)	47.41 ± 32.28 (50.00; 25.00–75.00)	0.73	−0.01
GSRS					
Diarrhea	2.11 ± 1.44 (1.67; 1.00–2.67)	2.03 ± 1.36 (1.67; 1.00–2.67)	2.43 ± 1.74 (1.67; 1.00–4.00)	0.59	−0.04
Indigestion	3.17 ± 1.29 (3.00; 2.25–4.00)	3.15 ± 1.18 (2.88; 2.25–4.00)	3.25 ± 1.70 (3.00; 1.75–4.50)	0.91	0.01
Constipation	2.75 ± 1.68 (2.33; 1.33–3.67)	2.72 ± 1.58 (2.33; 1.33–3.67)	2,87 ± 2.04 (2.00; 1.00–4.33)	0.74	0.03
Abdominal pain	2.55 ± 1.15 (2.33; 1.67–3.33)	2.54 ± 1.10 (2.33; 1.67–3.33)	2.59 ± 1.33 (2.33; 1.67–3.00)	0.85	0.01
Reflux	1.95 ± 1.36 (1.00; 1.00–2.50)	1.92 ± 1.34 (1.00; 1.00–2.50)	2.07 ± 1.49 (1.50; 1.00–2.50)	0.69	−0.03

^1^ Mann–Whitney test; ^2^ Glass rank biserial coefficient; LQD—low-pro-healthy-diet-quality group; MQD—medium-pro-healthy-diet-quality group; ThyPROpl—the Thyroid-specific Questionnaire; GSRS—the Gastrointestinal Symptom Rating Scale.

## Data Availability

The data are not publicly available due to internal regulations.

## Supplementary Materials

The following supporting information can be downloaded at: https://www.mdpi.com/article/10.3390/nu17061015/s1, Table S1. Summary of the components of the Healthy Diet Index—selected food items’ consumption frequency (times/day).
