# Supplementary material for: No Association Between Diet Quality, Nutritional Status, and Quality of Life in Women with Hashimoto’s Thyroiditis—A Cross-Sectional Study
Source: Nutrients. 2025 Mar 14;17(6):1015. doi: 10.3390/nu17061015 (PMC11944426; doi:10.3390/nu17061015)
Supplement: Supplementary file 1 [file nutrients-17-01015-s001.zip › nutrients-3489334-supplementary.pdf]

Supplementary Materials

Table S1. Summary of the components of the Healthy Diet Index—the frequency (times/day) of consumption of the selected food items.

Food groups/products	Total (n = 147) Mean ± SD (Median; Q1–Q3)	LQD (n = 118) Mean ± SD (Median; Q1–Q3)	MQD (n = 29) Mean ± SD (Median; Q1–Q3)	p-value [1]	Effect size [2]
Wholemeal bread/rolls	0.49 ± 0.52 (0.50; 0.06–0.50)	0.39 ± 0.44 (0.32; 0.06–0.5)	0.88 ± 0.65 (0.50; 0.50–1.00)	< 0.001	-0.34
Coarse-grained cereals	0.36 ± 0.38 (0.14; 0.06–0.50)	0.28 ± 0.29 (0.14; 0.06–0.50)	0.72 ± 0.49 (0.50; 0.50–1.00)	< 0.001	-0.41
Milk, flavored milk	0.82 ± 0.77 (0.50; 0.06–2.00)	0.69 ± 0.72 (0.50; 0.06–1.00)	1.33 ± 0.76 (2.00; 1.00–2.00)	< 0.001	-0.29
Fermented milk beverages	0.35 ± 0.34 (0.14; 0.06–0.50)	0.28 ± 0.29 (0.14; 0.06–0.50)	0.63 ± 0.38 (0.50; 0.50–1.00)	< 0.001	-0.38
Fresh cheese curd products	0.20 ± 0.23 (0.14; 0.06–0.50)	0.17 ± 0.19 (0.06; 0.06–0.14)	0.35 ± 0.29 (0.14; 0.14–0.50)	< 0.001	-0.29
White meat	0.35 ± 0.28 (0.50; 0.06–0.50)	0.35 ± 0.28 (0.50; 0.06–0.50)	0.35 ± 0.28 (0.50; 0.06–0.50)	0.93	0.01
Fish	0.13 ± 0.15 (0.06; 0.06–0.14)	0.12 ± 0.14 (0.6; 0.06–0.14)	0.17 ± 0.18 (0.06; 0.06–0.14)	0.18	-0.10
Pulse-based foods	0.17 ± 0.25 (0.06; 0.06–0.14)	0.15 ± 0.18 (0.06; 0.06–0.14)	0.26 ± 0.41 (0.06; 0.06–0.50)	0.25	-0.09
Fruit	0.89 ± 0.59 (1.00; 0.50–1.00)	0.77 ± 0.51 (0.50; 0.50–1.00)	1.41 ± 0.64 (2.00; 1.00–2.00)	< 0.001	-0.36
Vegetables	1.32 ± 0.68 (1.00; 0.50–1.00)	1.19 ± 0.67 (1.00; 0.50–2.00)	1.83 ± 0.45 (2.00; 2.00–2.00)	< 0.001	-0.34
pHDI-10 total score [pts.]	25.48 ± 9.59 (25.00; 19.10-32.70)	21.99 ± 6,89 (22.90; 16.90-27.0)	39.65 ± 4.69 (38.4; 36.3-42.0)	< 0.001	-0.69

[1] Mann-Whitney test; [2] Glass rank biserial coefficient; pHDI-10 - Pro-Healthy Diet Index-10; LQD—low-pro-healthy-diet-quality group; MQD—medium-pro-healthy-diet-quality group.
